# Generation of Rho Zero Cells: Visualization and Quantification of the mtDNA Depletion Process

**DOI:** 10.3390/ijms16059850

**Published:** 2015-04-30

**Authors:** Susanna Schubert, Sandra Heller, Birgit Löffler, Ingo Schäfer, Martina Seibel, Gaetano Villani, Peter Seibel

**Affiliations:** 1 Molecular Cell Therapy, Center for Biotechnology and Biomedicine (BBZ), Universität Leipzig, 04103 Leipzig, Germany; E-Mails: susanna.schubert@bbz.uni-leipzig.de (S.S.); sheller@tulane.edu (S.H.); birgit.loeffler@bbz.uni-leipzig.de (B.L.); ingo.schaefer@bbz.uni-leipzig.de (I.S.); gaetano.villani@uniba.it (G.V.); 2 Translational Centre for Regenerative Medicine (TRM), Universität Leipzig, 04103 Leipzig, Germany; 3 Department of Pathology and Laboratory Medicine, Tulane University, New Orleans, LA 70112, USA; 4 RhoZero Technologies, 97292 Uettingen, Germany; E-Mail: martina.seibel@rhozero.com; 5 Department of Basic Medical Sciences, Neurosciences and Sense Organs, University of Bari, 70124 Bari, Italy

**Keywords:** mitochondria, mitochondrial DNA (mtDNA), nucleoids, ρ^0^ cells, restriction endonuclease EcoRI, depletion system

## Abstract

Human mitochondrial DNA (mtDNA) is located in discrete DNA-protein complexes, so called nucleoids. These structures can be easily visualized in living cells by utilizing the fluorescent stain PicoGreen^®^. In contrary, cells devoid of endogenous mitochondrial genomes (ρ^0^ cells) display no mitochondrial staining in the cytoplasm. A modified restriction enzyme can be targeted to mitochondria to cleave the mtDNA molecules in more than two fragments, thereby activating endogenous nucleases. By applying this novel enzymatic approach to generate mtDNA-depleted cells the destruction of mitochondrial nucleoids in cultured cells could be detected in a time course. It is clear from these experiments that mtDNA-depleted cells can be seen as early as 48 h post-transfection using the depletion system. To prove that mtDNA is degraded during this process, mtDNA of transfected cells was quantified by real-time PCR. A significant decline could be observed 24 h post-transfection. Combination of both results showed that mtDNA of transfected cells is completely degraded and, therefore, ρ^0^ cells were generated within 48 h. Thus, the application of a mitochondrially-targeted restriction endonuclease proves to be a first and fast, but essential step towards a therapy for mtDNA disorders.

## 1. Introduction

The energy demand of eukaryotic cells is primarily covered by the ATP production of the oxidative phosphorylation system (OXPHOS) that is located in the inner membrane of the mitochondria. Genetically, this system is composed of proteins encoded in part by the nuclear and the mitochondrial genome. Only the coordinated genetic interplay of the genes of both genomes guarantees the proper function of OXPHOS.

In humans, genetic defects in nuclear or mitochondrial genes are known to cause severe deficiencies in oxidative energy supply. It is thought that in these patients the energy demand of the affected tissues cannot be solely compensated by the anaerobic energy generated during glycolysis. Ultimately, the decreased energy level cannot bolster the vital function of cells and tissues so that energy crisis becomes manifested causing a variety of clinical symptoms (e.g., blindness, dementia, autism, myalgia, disturbance of equilibrium [1]).

Giuseppe Attardi’s group introduced first in 1990 a remarkable approach to study mitochondrial disorders: by generating cells devoid of endogenous mtDNA (so called ρ^0^ cells), they were able to create a “mitochondrial acceptor” cell line that can be kept alive by applying special culture conditions (supplementation of growth media with uridine and pyruvate). These cells were subsequently used in fusion experiments with patient’s cytoplasts (cells without nuclei) to study patient’s mitochondria in an otherwise “normal” nuclear background of the acceptor cells [2].

The pitfall of this system can be identified as the part of the method that is used to generate the acceptor cells: by applying ethidium bromide to the growth medium over a period of 4 to 8 weeks, cells lose their mtDNA due to a reduced mtDNA replication rate [3]. However, disadvantageous mutagenic effects of ethidium bromide and other chemicals were reported (see [4,5,6] for further details). It cannot be excluded that the generation of ρ^0^ cells via these methods causes changes in the nuclear background, thus rendering the ρ^0^ cell’s nuclear background as potentially mutated.

To overcome this problem, we recently developed a method that takes advantage of an enzymatic approach to generate ρ^0^ cells: a modified restriction enzyme can be directed into mitochondria to cleave the endogenous genomes in more than two fragments depending on the cell’s haplotype background [7]. This event would then trigger endogenous mitochondrial nucleases to destroy completely the mitochondrial genome (for details see [8,9]). The basis of this system is the vector pMEE-con (depletion system with circular DNA). It was shown that the newly developed enzymatic strategy for mtDNA depletion is a convenient and suitable tool [8]. A depletion system obtained by PCR amplification of pMEE-con (MEE-con-module, depletion system with linear DNA) is also used to verify whether or not the structure of the depletion system plays a major role in the localization of EcoRI and the degradation of mitochondrial DNA.

In human mitochondria, discrete patches of DNA can be visualized by high-resolution electron or fluorescence microscopy, utilizing the DNA intercalating dyes ethidium bromide or DAPI as staining agents. These so-called nucleoids are made up of copies of the endogenous mitochondrial genome [10] as well as a yet not fully identified protein moiety. Most likely, the proteins belong to the mitochondrial transcription or replication machinery or take part in the localization of the mtDNA to the inner mitochondrial membrane [11,12,13,14,15]. Nucleoids have a uniform mean size of approximately 100 nm in mammals and they contain only a single copy of mtDNA (average approximately 1.4 mtDNA molecules per nucleoid) [16]. By utilizing Quant-iT™ PicoGreen^®^ [17] in conjunction with the restriction enzyme directed to mitochondria (mtDNA depletion system) we were able to visualize for the first time the ρ^0^ generation process. Mitochondrial nucleoids were resolved as early as 48 h post-transfection. The vanishing of the nucleoids already at that time underlines the extremely rapid mtDNA destruction caused by our method to generate ρ^0^ cells. The decrease of the relative mtDNA amount in a population of transfected/non-transfected cells and transfected cells after fluorescence-activated cell sorting was confirmed by real-time PCR analysis to further substantiate the qualitative analysis.

Therefore, ρ^0^ cells generated by this enzymatic method can definitely improve the utilization of cellular models in mitochondrial research.

## 2. Results and Discussion

The enzymatic degradation of mitochondrial DNA with a mitochondrially-targeted restriction endonuclease is an important first step towards the replacement of defective mitochondrial genomes and thus a genetic therapy.

It has been clear from previous experiments that the action of the restriction enzyme occurs soon after transfection [9]. In the present work we have been able to visualize this process *in vivo* by confocal fluorescence microscopy taking advantage of PicoGreen^®^, a fluorescent dye known to interact in a highly specific manner with DNA [17,18].

When cells were stained with PicoGreen^®^, cytoplasmic nucleoids appeared within the mitochondrial network of a cell as units of genetic inheritance [13,19], thereby indicating an uneven focal distribution of mtDNA molecules throughout the mitochondrial network. The shape, size and fluorescence intensity of the detected nucleoids in our study are consistent with previous findings [20]. Most likely, the nucleoids are either directly or indirectly attached to the inner mitochondrial membrane and are somehow associated with cytoplasmic tubulin and kinesin [14].

In our study we took advantage of the fact that the core structure of the nucleoids is made up of the mitochondrial genomes [10]. Hence, the destruction of the mtDNA by our enzymatic approach leads ultimately to the breakup of the nucleoid structure. When the number of nucleoids is taken as a rough measure for the integrity of mitochondrial DNA, the disappearance of the nucleoids indicates the degeneration of the endogenous mitochondrial genomes.

### 2.1. Visualization of Mitochondrial DNA Depletion Process

To visualize mitochondrial DNA depletion combined with the generation of ρ^0^ cells, microscopic and PCR-based methods were applied. The depletion systems pMEE-con and MEE-con-module lead to the expression of the restriction endonuclease EcoRI [9]. The import of EcoRI into the mitochondria is achieved with a mitochondrial targeting sequence (see Figure S1). Transfection efficiency and localization can be easily analyzed because the attached green fluorescent protein (EGFP) illuminates EcoRI paths of action. After transfection with the depletion system the mitochondrial localization of EGFP-EcoRI was confirmed.

**Figure 1:**
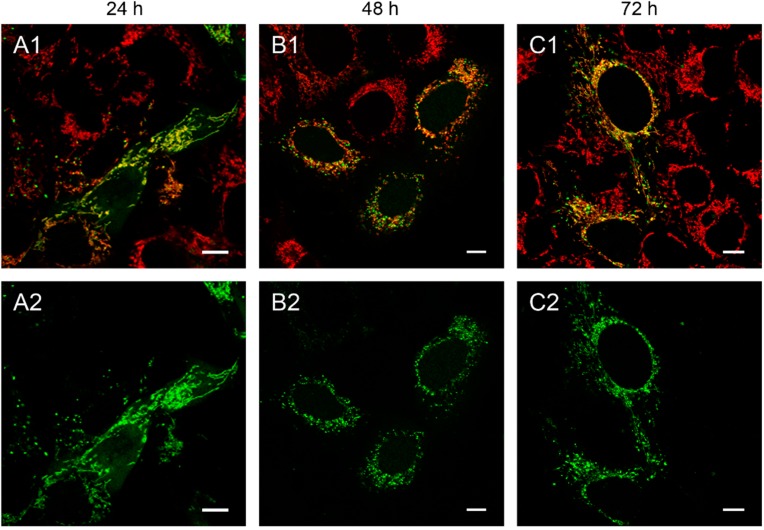
143B.TK^−^ cells transfected with linear depletion system. 143B.TK^−^ cells were transfected with the linear depletion system (MEE-con-module) and analyzed by confocal laser scanning microscopy. The EGFP-tagged restriction endonuclease (enhanced green fluorescent protein, green color, panels **A2**–**C2**) shows a uniform distribution or a punctate appearance (“nucleoid” structure) and co-localizes with the MitoTracker^®^ Red CMXRos-stained mitochondrial network (red color, panels **A1**–**C1**). The superimposition of both colors is depicted in the top panel. Images were collected at intervals of 24 h post-transfection. White arrows show dissolving mitochondrial network. Calibration marks correspond to 10 µm.

**Figure 2:**
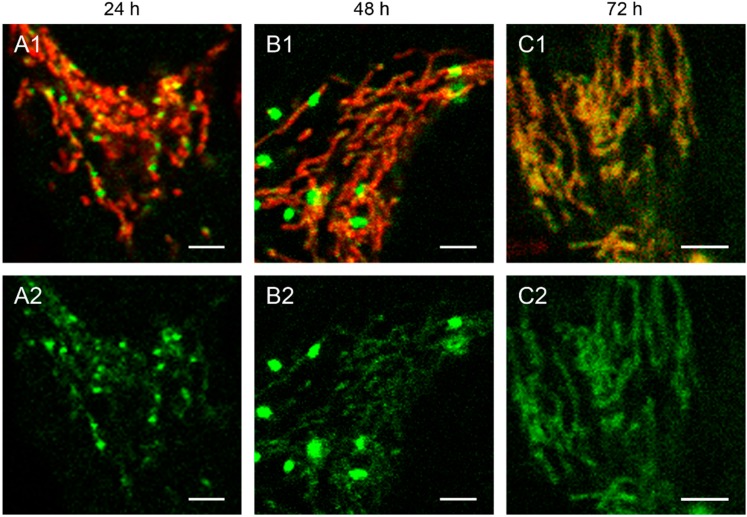
Detailed images of HEp-2 cells transfected with circular depletion system. Cells were transfected with the circular depletion system (pMEE-con with EGFP, green color, bottom panels **A2**–**C2**) and analyzed by confocal laser scanning microscopy at intervals of 24 h post-transfection. The mitochondrial network was stained with MitoTracker^®^ Red CMXRos (red color, overlay top panels **A1**–**C1**). The punctate appearance of the fusion protein EGFP-EcoRI merged into an evenly stained mitochondrial network 72 h post-transfection compared to 24 h/48 h, indicating that the interacting partner (mtDNA) of the restriction enzyme disappeared. Calibration marks correspond to 2.5 µm.

We observed that the mitochondrial localization of the fluorescently labeled restriction enzyme is associated with the destruction of mtDNA in the transfected cells. This becomes evident by overlaying the green EGFP fluorescence with the red staining of mitochondria with the specific dye MitoTracker^®^ Red CMXRos (Figure 1 and Figure 2). Transfection with linear and circular depletion system was carried out both in 143B.TK^−^ and HEp-2 cells, respectively.

At 24 h post-transfection the expression of the appropriate PCR product in 143B.TK^−^ cells (Figure 1A) lead firstly to an even distribution of EGFP-EcoRI fluorescence within mitochondria. Additionally, only few cells showed EGFP fluorescence in distinct sparkles, indicating possible destruction sites.

At 48 h post-transfection with the linear depletion system (Figure 1B), the mitochondrial matrix was not evenly stained. The clear-cut punctate staining differed remarkably from the tubular appearance of mitochondria as visualized by MitoTracker^®^ Red CMXRos staining. The superimposition of both images (Figure 1B1) underlines this observation, as demonstrated by the yellow sparkle appearance of the restriction enzyme in an otherwise red mitochondrial network. This indicates that the fluorescently tagged restriction enzyme localizes to distinct regions within the tubular network of mitochondria.

The observed punctate structure starts to dissolve in some cells into a tubular staining at 72 h post-transfection (white arrows), while others remain in a distinct localization within the tubular network (see Figure 1, last row).

The interaction of mitochondrial DNA with endonuclease EcoRI in HEp-2 cells is clearly shown in Figure 2, where three time points of the nucleoid structures up to the complete disappearance are displayed in detail.

When cells were transfected with the EGFP-labeled destruction system, an even distribution of the fusion enzyme could be seen in the mitochondrial network during the first hours. This is consistent with the assumption that the maturation (folding) of the EcoRI-EGFP fusion protein has to be completed before the enzyme can interact with its substrate (see Figure 1A). The image of a detailed HEp-2 cell at a higher magnification shows both the even distribution and the punctate structure at 24 h post-transfection (Figure 2A). At 48 h post-transfection, nucleoids became more visible indicating, that the fusion proteins were mainly associated with their substrates in order to start the cleavage procedure of the genomes (Figure 2B). At 72 h the green staining started to disperse throughout the mitochondrial network thus overlaying with the MitoTracker^®^ Red staining (Figure 2C).

The moment of a punctate structure appearance followed by an even distribution depends on different factors: the maturation of the protein, the binding of EcoRI to the mtDNA and the utilized cell line. In line with our previous finding [9], mtDNA depletion could usually be detected generally as early as 48 h post-transfection, independently of the used depletion system (circular or linear).

To further challenge the hypothesis that mtDNA is degraded by the EGFP-EcoRI co-localized with the nucleoid structure, transfected cells were stained with the fluorescent dye PicoGreen^®^ that by intercalating double stranded DNA visualizes nuclear and mitochondrial DNA [17,18]. The applied settings for confocal laser scanning microscopy imaging allowed the preferential detection of the nucleoid structure and nuclear staining by PicoGreen^®^ as the EGFP-EcoRI signal is hidden by the brightness of PicoGreen^®^ at a similar excitation and emission wave length.

**Figure 3:**
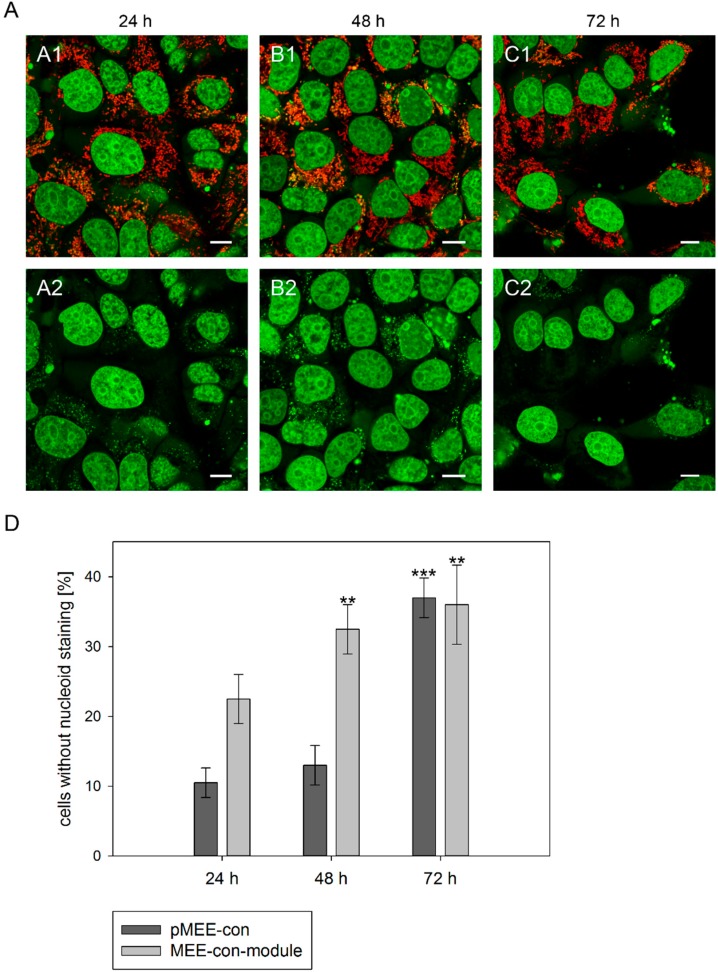
Analysis of nucleoid staining in 143B.TK^−^ cells. (**A**) Confocal images. 143B.TK^−^ cells transfected with pMEE-con were analyzed by microscopy. The nucleus and nucleoids are stained green (PicoGreen^®^; panels **A2**–**C2**). The mitochondrial network was stained with MitoTracker^®^ Red CMXRos and co-localizes with green-stained nucleoids (panels **A1**–**C1**, overlay). A few cells do not show nucleoid staining (indicating ρ^0^ status). Calibration marks correspond to 10 µm; (**D**) Quantification of images. More than one hundred 143B.TK^−^ cells were counted and analyzed. The diagram shows the percentage of cells without nucleoid staining after transfection with various depletion systems. Bars describe the arithmetic mean ± SD. ** *p* < 0.01, *** *p* < 0.001.

Therefore, 143B.TK^−^ cells were transfected with the circular depletion system and incubated with MitoTracker^®^ Red CMXRos and PicoGreen^®^ dsDNA reagent. As expected, punctate staining indicating mitochondrial nucleoids was visualized in most cells at 24 h post initiation process (see Figure 3A, first row) with only few individual cells showing exclusively nuclear staining. The number of cells without punctate green structures increased over time, indicating that the nucleoid structures dissolve (Figure 3A, 48 and 72 h). To highlight these results a control is depicted in Figure S2, cells without transfection and incubation time (0 h). Similar data were obtained when transfecting 143B.TK^−^ cells with the linear depletion system (MEE-con-module).

In order to quantify the above observation, more than one hundred 143B.TK^−^ cells (stained with MitoTracker^®^ Red CMXRos and PicoGreen^®^) were counted for each field and analyzed at every time point (Figure 3D). After the transfection with the depletion systems, approximately 30% of the cells had lost their nucleoid staining after 72 h.

**Figure 4:**
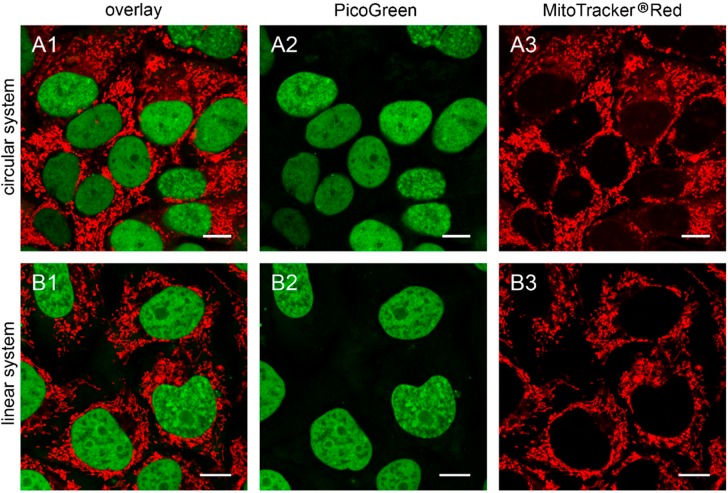
The 143B.TK^−^ ρ^0^ K7 cells transfected with depletion systems. The 143B.TK^−^ ρ^0^ K7 cells were transfected with pMEE-con (**A**) and MEE-con-module (**B**) and analyzed 24 h post-transfection by confocal laser scanning microscopy. Cells were incubated with PicoGreen^®^ (green color, panel **A2**,**B2**) to stain DNA. The mitochondrial network was stained with MitoTracker^®^ Red CMXRos (red color, panel **A3**,**B3**). Overlay of both fluorescent stainings are depicted in panel **A1** and **B1**. Calibration marks correspond to 10 µm.

To further confirm our observations, we stained established ρ^0^ cell lines with PicoGreen^®^. We analyzed 143B.TK^−^ ρ^0^ cells, previously generated by transfection with pMEE-con and cloning [9], after additional transfection with pMEE-con and MEE-con-module. As expected, the cells displayed only nuclear but no cytoplasmic punctate staining (Figure 4).

Similar results were obtained in HEp-2 ρ^0^ EtBr cells (generated by incubation with ethidium bromide) and HEp-2 ρ^0^ K1 cells (generated by transfection with pMEE-con). Therefore the absence of mitochondrial nucleoids after staining with PicoGreen^®^ is an indicator for ρ^0^ cells.

Additionally, it is worth noting that the absence of PicoGreen^®^ stained mitochondrial nucleoids in transfected or established ρ^0^ cells excludes the existence of DNA, either re-ligated or residual mtDNA molecules.

### 2.2. Quantitative Analysis of Mitochondrial DNA

**Figure 5:**
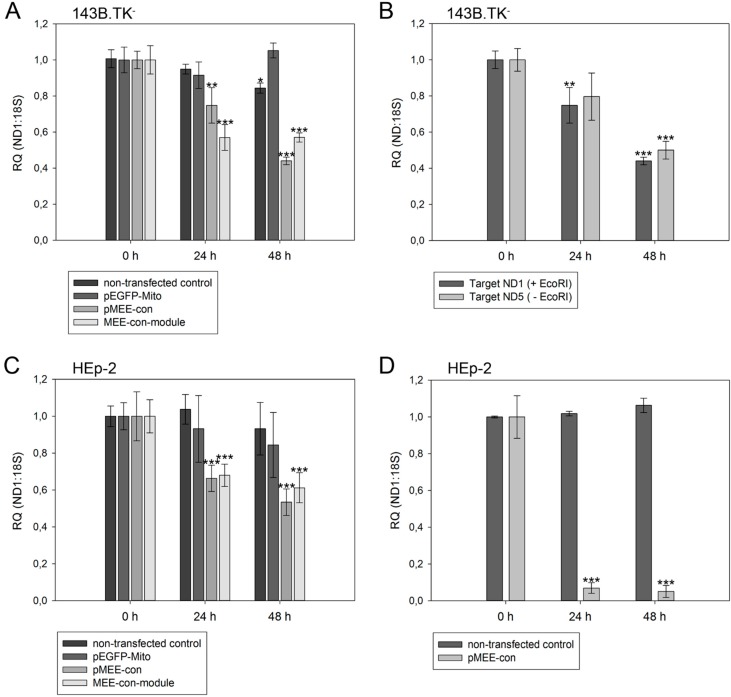
Relative quantification of mtDNA in transfected cells. Cells were harvested directly before (0 h) or 24 h/48 h post-transfection with pEGFP-Mito (transfection control), circular depletion system (pMEE-con) or linear depletion system (MEE-con-module). Furthermore, non-transfected cells were used as control. Experiments were performed in triplicate. Data are expressed as ratio between mitochondrial DNA (*ND1* or *ND5* gene) *vs.* nuclear (ribosomal *18S* gene) DNA content. Bars represent the mean ± SD. * *p* < 0.05, ** *p* < 0.01, *** *p* < 0.001. (**A**) 143B.TK^−^ cells; (**B**) 143B.TK^−^ cells transfected with depletion system (pMEE-con). Relative mtDNA content of DNA isolates was analyzed using *ND1* (04094–04175 bp) and *ND5* (13,893–13,983 bp) gene as targets in real-time PCR. Amplified target sequences differ in encompassing a recognition site for the restriction endonuclease EcoRI; (**C**) HEp-2 cells; (**D**) HEp-2 transfected cells. EGFP expressing cells were separated from non-transfected cells by fluorescence-activated cell sorting.

To challenge the hypothesis that mtDNA is fully depleted by endogenous nucleases after transfection with our kit, a quantitative analysis of mtDNA in a transfected cell population was performed by real-time PCR. As shown in Figure 5, the relative amount of mtDNA (*ND1* or *ND5* genes) *vs.* nuclear DNA (18S ribosomal gene) was measured using different primer pairs both in 143B.TK^−^ and HEp-2 cells. The amplification of nuclear mitochondrial DNA fragments was largely excluded (see Supplementary Materials Section 2, Figure S3) [21,22,23,24,25,26].

Firstly, to rule out any negative effect of the transfection procedure and/or of the recombinant protein expression on mitochondrial DNA content, cells were transfected with a construct for the expression of mitochondrially targeted EGFP without nucleolytic activity (pEGFP-Mito). Transfected cells did not show any significant variation of mtDNA content (Figure 5A,C, gray bars), thus excluding any unspecific effect by the experimental procedures on mtDNA content. The transfection efficiency 48 h post-transfection was approximately 45% for 143B.TK^−^ cells and 60% for HEp-2 cells, respectively.

Next, cells were transfected with the depletion systems and the DNA was quantified utilizing real-time PCR to prove that mtDNA is degraded after transfection.

As expected, a significant reduction of relative mtDNA content in 143B.TK^−^ cells was observed 24 h post-transfection with the depletion kit: the total mtDNA amount was reduced by 25% (pMEE-con) and 40% (MEE-con-module) (see Figure 5A). At 48 h post-transfection with the circular depletion kit, the observed 55% reduction of mtDNA was in line with the 45% transfection efficiency, thus suggesting that total loss of mtDNA occurred in transfected cells. Interestingly, the data also show that the mtDNA depletion is less effective when using the linearized pMEE-con transfection (Figure 5A, light gray bars). Again, the mtDNA decrease (30%) was consistent with the transfection efficiency (30%) thus pointing to a lower uptake of linearized DNA during transfection [27]. Furthermore, the presence of a similar mtDNA content 24 and 48 h post-transfection with the linear depletion system could be explained by the lack of origin of replication and the consequent dilution of the construct during cell division. These findings were confirmed by the results obtained with the HEp-2 cell line (Figure 5C, light gray bars).

To exclude the contribution of non-transfected cells in the quantitative analysis, a fluorescence-activated cell sorting was used to separate the two populations of cells based on the expression of the EGFP fusion protein. HEp-2 cells transfected with the circular depletion system were sorted by flow cytometry and mtDNA content was measured by quantitative real-time PCR (Figure 5D). In comparison to non-transfected parental cells the mtDNA content in sorted cells was drastically decreased to 7% and 5% at 24 and 48 h post-transfection showing that the circular depletion vector could reach its maximum efficiency already at 24 h post-transfection.

Specific destruction of mtDNA mutated molecules associated with mitochondrial diseases has been proposed to be a potential therapy for mitochondrial diseases caused by mutations that create a unique restriction endonuclease cleavage site in the mtDNA [28,29]. In contrast to these studies we offer with other methods a more rapid destruction of total mtDNA with the restriction endonuclease EcoRI to generate ρ^0^ cells, as EcoRI creates three to five destruction start sites, depending on the mtDNA background. However, it is well understood that a repopulation with intact mitochondrial genomes is an essentially required second step when thinking of a genetic therapy (patent pending, [30]).

With our depletion strategy, after cleavage of mtDNA, endogenous nucleolytic enzymes start to degrade DNA at the generated 5'- and 3'-endings. To distinguish the cleaving and degrading process of mtDNA in 143B.TK^−^ cells after expression of mitochondrially targeted EGFP-EcoRI, mtDNA depletion was assessed using two different primer sets. The first set hybridized to the mitochondrial *ND1* gene thereby encompassing an EcoRI restriction site. Hence, only un-cleaved mtDNA molecules would be amplified (Figure 5B, dark gray bars). A second primer set, hybridizing to the mitochondrial *ND5* gene that does not contain an EcoRI recognition site was taken. This set can amplify mtDNA that is cleaved but not completely degraded by endogenous enzymes (Figure 5B, light gray bars). The data show that both mtDNA species can be amplified to a similar extent in a transfected cell population over a period of 48 h, thus suggesting that degradation of mtDNA by endogenous nucleases occurs soon after cleavage.

## 3. Experimental Section

### 3.1. DNA

The plasmid pMEE-con is a fusion gene product consisting of the endonuclease EcoRI gene and a mitochondrial targeting sequence derived from the human cytochrome c oxidase subunit 8 (please see Figure S1 and [9] for details). After transfection and constitutive expression of restriction endonuclease EcoRI, the EGFP green fluorescent marker could monitor the mitochondrial localization of the fusion protein. The circular depletion vector (pMEE-con) was used directly for transfection experiments and as template for PCR utilizing the following primers: pEGFP-N1-1868-REV 5'-GGGCCATCGCCCTGATAG-3' and pTRE2hyg-2364-REV 5'-AGTTAGGCCACCACTTCAAGAACTCT-3'. In addition to this experiment, the PCR-product was used for transfection as linear depletion system (MEE-con-module).

The control vector pEGFP-Mito leads to the expression of a mitochondrially targeted EGFP.

### 3.2. Cell Culture

Human osteosarcoma cells 143B.TK^−^ (ATCC CRL-8303) were grown in high glucose Dulbecco’s modified Eagle’s medium (DMEM, with stable glutamine and pyruvate) supplemented with 10% (*v*/*v*) fetal calf serum (FCS) and 100 μg/mL bromodeoxyuridine (BrdU), at 37 °C in a humidified atmosphere containing 5% CO_2_.

Human larynx carcinoma cells HEp-2 (ATCC CCL-23) were cultured under standard conditions (37 °C, 5% CO_2_) in DMEM (high glucose, with stable glutamine and pyruvate) supplemented with 10% (*v*/*v*) FCS and 100 µM non-essential amino acids.

The ρ^0^ cell lines 143B.TK^−^ ρ^0^ K7 and HEp-2 ρ^0^ K1 were generated by transfection of wild type cells with pMEE-con (as described in [9]). The ρ^0^ cell line referred to as 143B.TK^−^ ρ^0^ EtBr and HEp-2 ρ^0^ EtBr were generated by incubation of wild type cells with low doses of ethidium bromide (as described in [3]). The ρ^0^ cell lines were maintained as indicated with additional supplementation of 50 µg/mL uridine.

### 3.3. Transfection and Labeling of Cells

For microscopic analysis, live cells were cultured for one day on glass bottom dishes (MatTek Corporation, Ashland, MA, USA). Transient transfection with circular (vector pMEE-con) and linear depletion system (MEE-con-module) were performed using *Trans*IT^®^-LT1 (Mirus, Madison, WI, USA) according to the manufacturer’s conditions. The transfected cells were cultured in media supplemented with uridine.

Cells were stained with MitoTracker^®^ Red CMXRos (Invitrogen, Molecular Probes, Karlsruhe, Germany) for mitochondrial network analysis. To stain nucleus and nucleoids, the cells were incubated with Quant-iT™ PicoGreen^®^ dsDNA reagent (Invitrogen) as described elsewhere [17].

For real-time PCR analysis the cells were cultured for one day on 35 mm culture dishes (TPP, Techno Plastic Products AG, Trasadingen, Switzerland) and subsequently were transfected as described before. An additional transfection control was carried out using a vector construct that leads to the expression of mitochondrially targeted EGFP (pEGFP-Mito).

### 3.4. Confocal Microscopy

The cells were analyzed with an inverted confocal laser scanning microscope TCS SP5 (Leica Microsystems, Wetzlar, Germany) after 24, 48 and 72 h of cultivation.

To avoid a cross talk in excitation of 488 and 561 nm multiple stained compounds, a sequential scanning mode was executed. Images were acquired with photomultipliers and micrographs were processed and analyzed with the software Leica Application Suite Advanced Fluorescence 2.6.0 (Leica Microsystems, Wetzlar, Germany), Adobe Photoshop CS (Adobe Systems, Munich, Germany), ImageJ 1.45s (NIH, Bethesda, MD, USA) and Huygens Professional 4.2.1 (SVI, Hilversum, The Netherlands).

### 3.5. Fluorescence-Activated Cell Sorting (FACS)

Transfected cells at each time point were sorted by flow cytometry (FACSAria SORP–cell sorter, BD Biosciences, Heidelberg, Germany). To detect the green fluorescent cells an argon-laser with 488 nm (TCS SP5 Leica Microsystems, Wetzlar, Germany) was utilized. The efficiency averaged over 95%. Harvested cells were directly processed for DNA isolation or stored at −20 °C.

### 3.6. Real-Time PCR

The loss of endogenous mitochondrial DNA (mtDNA) after transfection with the depletion systems was controlled by quantitative real-time PCR. Therefore, genomic DNA (nDNA) of parental, transfected and sorted cells were isolated via phenol/chloroform extraction at different time points after transfection (0, 24 and 48 h). Relative quantification of mtDNA and nDNA was carried out employing a 7500 real-time PCR system from Applied Biosystems (Weiterstadt, Germany), software SDS V1.2.3 and the qPCR MasterMix Plus for SYBR^®^ Green I Low ROX (Eurogentec, Seraing, Belgium). As endogenous control the *18S* gene was used (*18S*-1036-FW 5'-AGTCGGAGGTTCGAAGACGAT-3' and *18S*-1127-REV 5'-GCGGGTCATGGGAATAACG-3'). The mitochondrial target gene *ND1* (04094-FW 5'-CCCTACTTCTAACCTCCCTGTTCTTAT-3' and 04175-REV 5'-CATAGGAGGTGTATGAGTTGGTCGTA-3', flanking an EcoRI recognition site) or the mitochondrial *ND5* gene (13893-FW 5'-ATTTTATTTCTCCAACATACTCGGATT-3' and 13983-REV 5'-GGGCAGGTTTTGGCTCGTA-3') were utilized for the relative evaluation of mtDNA compared to nDNA of cells post-transfection.

The analysis of mtDNA in ρ^0^ cells and parental cell lines was carried out by quantitative real-time PCR (Supplementary Materials) after isolation of DNA (software SDS V1.5.1). In this case the Takyon Low ROX SYBR MasterMix blue dTTP (Eurogentec, Seraing, Belgium) was used because of manufacturer’s conversion. Different endogenous controls were employed: *18S* gene (*18S*-1036-FW and *18S*-1127-REV); *β-Actin* gene (*ACTB*-617-FW 5'-GGGAAATCGTGCGTGACATTA-3' and *ACTB*-762-REV 5'-CCGCTCATTGCCAATGGT-3') and *COX6A1* gene (*COX6A1*-099-FW 5'-CTCAGCTCGCATGTGGAAGA-3' and *COX6A1*-244-REV5'-TGGTCCTGATGCGGAGATG-3'). To detect mtDNA fragments following primer sets were used: *ND1* (03497-FW 5'-CCACATCTACCATCACCCTC-3' and 03565-REV 5'-TTCATAGTAGAAGAGCGATGGT-3', without EcoRI recognition site), *ND1* (04094-FW and 04175-REV, encompass EcoRI recognition site); *ND2* (04841-FW 5'-GACATCCGGCCTGCTTCTT-3' and 04922-REV 5'-TACGTTTAGTGAGGGAGAGATTTGG-3'); *ND5* (12574-FW 5'-TTCAAACTAGACTACTTCTCCATAATATTCATC-3' and 12674-REV 5'-TTGGGTCTGAGTTTATATATCACAGTGA-3', containing EcoRI recognition site) and ND5 (13893-FW and 13983-REV, without EcoRI recognition site).

### 3.7. Statistical Analysis

Data of relative mtDNA quantification are shown as mean ± standard deviation (SD) of two or three identical experiments performed in triplicate. To analyze the statistically significant differences between non-transfected and transfected cells an unpaired two-sample *t*-test was rendered applying software SigmaPlot version 12.0 (Systat Software, Erkrath, Germany). Differences were designated significant at values * *p* < 0.05, highly significant at ** *p* < 0.01 and very highly significant at *** *p* < 0.001 and were labeled with asterisks.

The qualitative changes of cells with or without nucleoids were counted using the acquired confocal images. A minimum of one hundred cells per sample was counted. Because an average of only two values was analyzed, statistical significance could not be determined.

## 4. Conclusions

In this study we demonstrated for the first time that the action of the mitochondrial DNA depletion system is immediate. We shed light on the time line of the process of mtDNA degradation by confocal fluorescence microscopy and furthermore by real-time PCR of mixed (transfected and non-transfected) and sorted cells, respectively.

The destruction of mitochondrial DNA can be easily followed by the disappearance of mitochondrial nucleoids. The time required to completely dissolve the mitochondrial nucleoids and thus the mitochondrial genomes can vary in a cell type as well as in an individual cell dependent manner. These experiments indicate that mtDNA depleted cells can be seen as early as 48 h post-transfection. A total of approximately 30% of nucleoids depleted cells in a transfected cell population were seen at 72 h post initiation process.

The cleavage of mtDNA was quantified by real-time PCR. The experiments could depict that cleaved mtDNA is subsequently degraded within 24 to 48 h post-transfection in different cell lines and with both depletion systems. Cell quantification after transfection and sorting reveals a drastic reduction of mtDNA content below 10%. Consequently the efficiency of the depletion system can be as high as 90% (depending on the cell line used).

Both technologies used to analyze the process of mtDNA degradation, confocal microscopy and real-time PCR, complement one another. Thus, the developed technology with the restriction endonuclease EcoRI allows a mild and very fast strategy to deplete endogenous genomes from cells so that these cells can be easily utilized in human cytogenetic experiments and diagnostics.

## Supplementary Materials

Supplementary materials can be found at http://www.mdpi.com/1422-0067/16/05/9850/s1.
