# Supplementary material for: Generation of Rho Zero Cells: Visualization and Quantification of the mtDNA Depletion Process
Source: Int J Mol Sci. 2015 Apr 30;16(5):9850–65. doi: 10.3390/ijms16059850 (PMC4463621; doi:10.3390/ijms16059850)
Supplement: Supplementary file 1 [file ijms-16-09850-s001.pdf]

Supplementary Information

1. Visualization of Mitochondrial DNA Depletion Process

The depletion systems pMEE-con and MEE-con-module lead to the expression of the restriction endonuclease EcoRI [9]. The import of EcoRI into the mitochondria is achieved with a mitochondrial targeting sequence (see Figure S1). Transfection efficiency and localization can be easily analyzed because of the attached green fluorescent protein (EGFP).

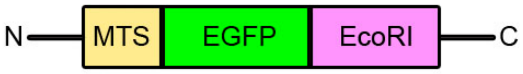

Figure S1. Organization of the mitochondrially targeted EcoRI. A DNA fragment coding for a mitochondrial targeting sequence (MTS; COX VIII) was added to the 5' end of the enhanced green fluorescent protein EGFP as optical marker and the EcoRI gene was fused to the 3' end of EGFP. The construct was cloned resulting in the vector pMEE-con with a constitutive CMV promoter.

To underline the results in Figure 3, wild type cells of 143B.TK^{-} were visualized as control without incubation time (0 h).

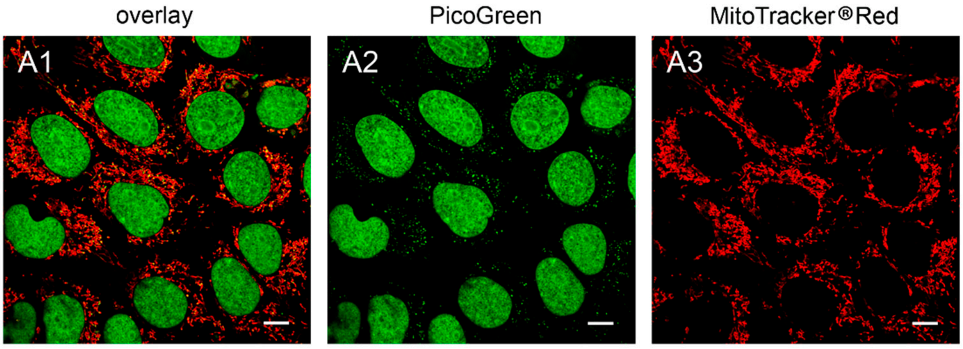

Figure S2. 143B.TK^{-} wild type cells. The control cells were stained with MitoTracker® Red CMXRos (red color) and PicoGreen® (green color). Calibration marks correspond to 10 µm.

2. Estimation of Nuclear mtDNA Fragments Amplification

The natural transfer of DNA from mitochondria to the nucleus during evolution has generated nuclear copies of mtDNA, the so-called *numts* [23]. The numbers of human *numts* was reported with various values ranging from 286 to 612 [21,24,26]. In order to overcome the signal interference due to *numts*, 143B.TK^{-} wild type and ρ^0 cells were analyzed by real-time PCR using different mtDNA primer sets in conjunction with the single copy gene *β-Actin* (Figure S3). For this purpose, the nuclear gene *Cox6A*1 and the two mitochondrial *ND1* and *ND5* genes were amplified by two primer sets, with one encompassing the restriction site of EcoRI and the other not containing the same site. Additionally the *ND2* gene was also detected with another primer set.

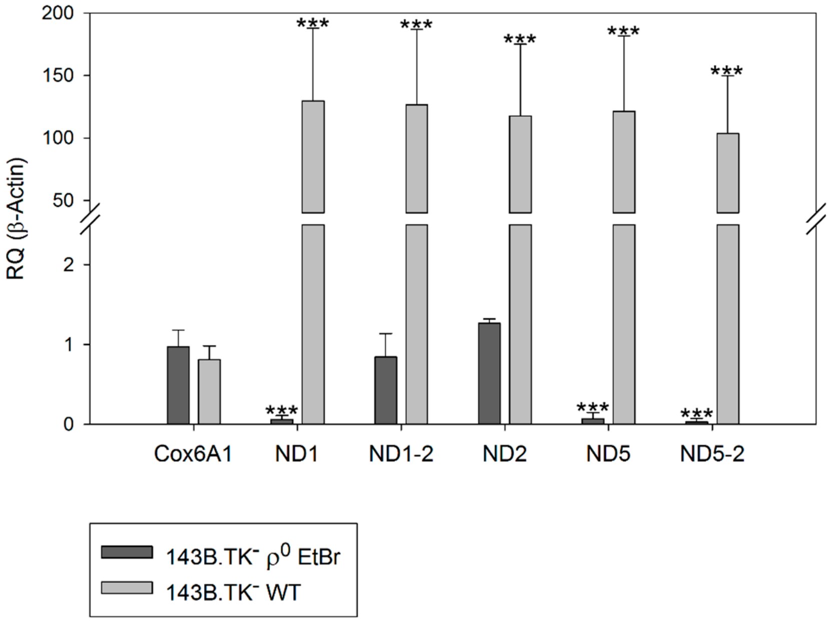

Figure S3. Analysis of 143B.TK$^-$ ρ^0 and wild type cells. Quantification of relative mtDNA content of ρ^0 cells in comparison to wild type (WT) cells. Mitochondrial primer sets for *ND1*, *ND2* and *ND5* were used. Relative mtDNA and *Cox6A1* gene content were normalized to nuclear *β-Actin* gene. The primer sets amplify mtDNA in given ranges: *ND1*: 03497–03565 bp; *ND1-2*: 04094–04175 bp; *ND2*: 04841–04922 bp; *ND5*: 12,574–12,674 bp; *ND5-2*: 13,893–13,983 bp. Experiments were performed in duplicate and triplicate. Bars present the mean ± SD. *** $p < 0.001$.

Conspicuously, several mtDNA fragments were detected in ρ^0 cells (see Figure 6, dark gray bars). The first *ND1* primer set in the mtDNA region of 3500 bp is amplified rarely in real-time PCR. In contrast, the second *ND1* fragment in region of 4000 bp (*ND1-2*) is detected with approximately one copy per *β-Actin* in 143B.TK$^-$ ρ^0 cells. The *ND2* gene shows similar values compared to the second *ND1* gene section. Consequently, the mtDNA fragments in the region between 4000 and 5000 bp are inserted in the nuclear DNA. This hypothesis could be confirmed by BLAST searches in the NCBI database and given literature [22,25].

In addition to *ND1* and *ND2* gene amplification, two *ND5* regions were analyzed under similar conditions. Both primer sets show only a slight amplification. Thus, this region of mtDNA is not inserted in nuclear DNA or inserted fragments differ quite significantly to mtDNA.

Real-time PCR was performed with the parental cell line 143B.TK$^-$ in a manner similar to that of ρ^0 cells, (Figure S3, light gray bars). It is notable that all investigated mtDNA fragments were detected in an approximately 150-fold amplification.
